# New island record and conservation status of Puerto Rican Bank endemic plant species, *Ruehssia woodburyana* (Acev.-Rodr.) Goyder, comb. nov., formally transferred from *Marsdenia*

**DOI:** 10.3897/BDJ.8.e47110

**Published:** 2020-01-23

**Authors:** Sara Bárrios, José A Sustache, David Goyder, Martin A Hamilton

**Affiliations:** 1 Royal Botanic Gardens, Kew, London, United Kingdom Royal Botanic Gardens, Kew London United Kingdom; 2 Department of Natural and Environmental Resources of Puerto Rico, San Juan, Puerto Rico Department of Natural and Environmental Resources of Puerto Rico San Juan Puerto Rico

**Keywords:** Apocynaceae, Caribbean Flora, Conservation, Endemism, New combination, Nomenclature, Red List

## Abstract

**Background:**

Thought to be endemic to the Commonwealth of Puerto Rico, *Ruehssia woodburyana* (Apocynaceae) was recently discovered at a single location on Norman Island in the British Virgin Islands. Despite an increase in the extent of occurrence and area of occupancy, this species meta-population is very limited with a total of 37 individuals known in the wild. The largest subpopulation, on Mona Island, has only 26 individuals. The species suitable habitat is experiencing a continuing decline due to urban development, grazing by feral ungulates and human-induced forest fires. Conservation action is urgently needed and should be directed towards establishing genetically representative *ex situ* collections, such as seed for long term storage and live material for propagation. This species is evaluated as Critically Endangered (CR), based on Criteria C2a(i)+D, according to the IUCN Red List Categories and Criteria (version 3.1) and guidelines (Subcommittee IUCN Standards and Petitions 2016).

**New information:**

Extensive and regular surveys to the region enable the discovery of new plant records for different countries and islands. In this paper, we record a new island record for *Ruehssia woodburyana* on Norman Island, in the British Virgin Islands and discuss the species conservation status. *Marsdenia woodburyana* is transferred to the genus *Ruehssia* to reflect the resurrection of that genus for species of *Marsdenia* native to the New World.

## Introduction

In this paper, we present a species conservation profile for an endemic species to the British Virgin Islands and to the Commonwealth of Puerto Rico.

## Species Conservation Profiles

### Ruehssia woodburyana

#### Species information

Scientific name: Ruehssia woodburyana

Species authority: (Acev.-Rodr) Goyder

Synonyms: *Marsdenia woodburyana* Acev.-Rodr., 1999 – Acevedo-Rodriguez 1999: 167.

Kingdom: Plantae

Phylum: Tracheophyta

Class: Magnoliopsida

Order: Gentianales

Family: Apocynaceae

Taxonomic notes: All the native New World species of the broadly delimited pan-tropical genus *Marsdenia* R.Br. (Apocynaceae: Asclepiadoideae) occur in a single clade, according to a recent study using two plastid and two nuclear gene regions (Espírito Santo et al. 2019). The genus *Ruehssia* H.Karst. was resurrected for these species but, to date, only those taxa occurring in Brazil have been transferred, although Cuban taxa will follow shortly (Liede-Schumann pers. comm. 2019, manuscript under review) and it is planned to transfer species from other parts of the Americas in subsequent papers.In order to expedite the range extension of *M. woodburyana* Acev.-Rodr. to the British Virgin Islands and to permit timely publication of its conservation status, we here propose the formal transfer of this species from *Marsdenia* to *Ruehssia*.***Ruehssia woodburyana*** (Acev.-Rodr.) Goyder, **comb. nov.**BASIONYM: *Marsdenia woodburyana* Acev.-Rodr., 1999 – Acevedo-Rodriguez 1999: 167.TYPE: Puerto Rico, Mun. Guánica, Bosque Estatal de Guánica, Caña Gorda, 26 May 1998, Ramírez & Rosado 27, holotype: US [US00604132]; isotypes: MAPR; MO [MO-169808]; NY [NY00328790]; UPRRP; US (alcohol collection)).

Region for assessment: Global

#### Editor & Reviewers

##### Reviewers

Reviewers: Clubbe, C.; Urbaniak, J.

##### Editor

Editor: Barrios, S.; Hamilton, M.A.

#### Reviewers

Reviewers: Clubbe, C.; Urbaniak, J.

#### Editor

Editor: Barrios, S.; Hamilton, M.A.

#### Geographic range

Biogeographic realm: Neotropical

Countries: Virgin Islands, BritishPuerto Rico

Map of records (image): 

Map of records (Google Earth): 

Basis of EOO and AOO: Observed

Basis (narrative): Observed occurrences.

Min Elevation/Depth (m): 10

Max Elevation/Depth (m): 150

Range description: *Ruehssia woodburyana* is a rare plant species restricted to the Commonwealth of Puerto Rico and the British Virgin Islands (BVI). This species was originally described occurring exclusively at Caña Gorda within the Guánica State Forest in Guánica municipality on the island of Puerto Rico (Acevedo-Rodriguez 1999, Acevedo-Rodríguez 2005). Herbarium collections and observations dating from 2007 to 2017 (Suppl. material 1) reveal that this species also grows in other southern coastal municipalities of Puerto Rico: Peñuelas, Lajas and Cabo Rojo (Segarra et al. 2014). There are also herbarium collections from the islands of Mona and Culebra in the Commonwealth of Puerto Rico. In 2017, a team of botanists from the Royal Botanic Gardens Kew, the National Parks Trust of the Virgin Islands, US Fish and Wildlife Service, the Department of Natural and Environmental Resources of Puerto Rico and the University of Puerto Rico (Mayagüez Campus) came across fertile material of a similar vine on Norman Island, an island on the south-western edge of the British Virgin Islands (BVI) archipelago (Hamilton and Barrios 2017). This vine was vouchered (M.A. Hamilton, #1738, K000860152, K!) and later confirmed as *R. woodburyana* (O. Monsegur pers. comm. 2017). This species extent of occurrence (EOO) was estimated to be 5,649 km^2^ and the area of occupancy to be 32 km^2^ based on a 2×2 km cell size (Bachman et al. 2011).

#### New occurrences

#### Extent of occurrence

EOO (km2): 5649

Trend: Increase

Causes ceased?: Unknown

Causes understood?: Unknown

Causes reversible?: Unknown

Extreme fluctuations?: No

#### Area of occupancy

Trend: Increase

Causes ceased?: Unknown

Causes understood?: Unknown

Causes reversible?: Unknown

Extreme fluctuations?: No

AOO (km2): 32

#### Locations

Number of locations: 7-8

Justification for number of locations: The number of locations was calculated to be seven to eight, considering threats posed by human disturbance and human-induced fires at the different sites where the species has been recorded.

Trend: Unknown

Extreme fluctuations?: No

#### Population

Number of individuals: 37

Trend: Unknown

Causes ceased?: Unknown

Causes understood?: Unknown

Causes reversible?: Unknown

Extreme fluctuations?: Unknown

Population Information (Narrative): Originally described from Guánica State Forest as extremely rare, Acevedo-Rodriguez (1999) mentioned a single mature plant and two juveniles. More recent collections from southern municipalities on the island of Puerto Rico, Culebra and Mona islands in the Commonwealth of Puerto Rico and Norman Island in the BVI suggest a total of 37 individuals. The largest sub-population occurs on Mona Island with 26 known individuals. All other subpopulations have between one and five individuals. The area and quality of suitable habitat of this species is in continuing decline due to grazing by feral ungulates and human disturbance, including development and human-induced fires.

#### Subpopulations

Abundance largest subpopulation: 26

Number of subpopulations: 4

Trend: Unknown

Extreme fluctuations?: No

Severe fragmentation?: No

#### Habitat

System: Terrestrial

Habitat specialist: Unknown

**Figure 1.**
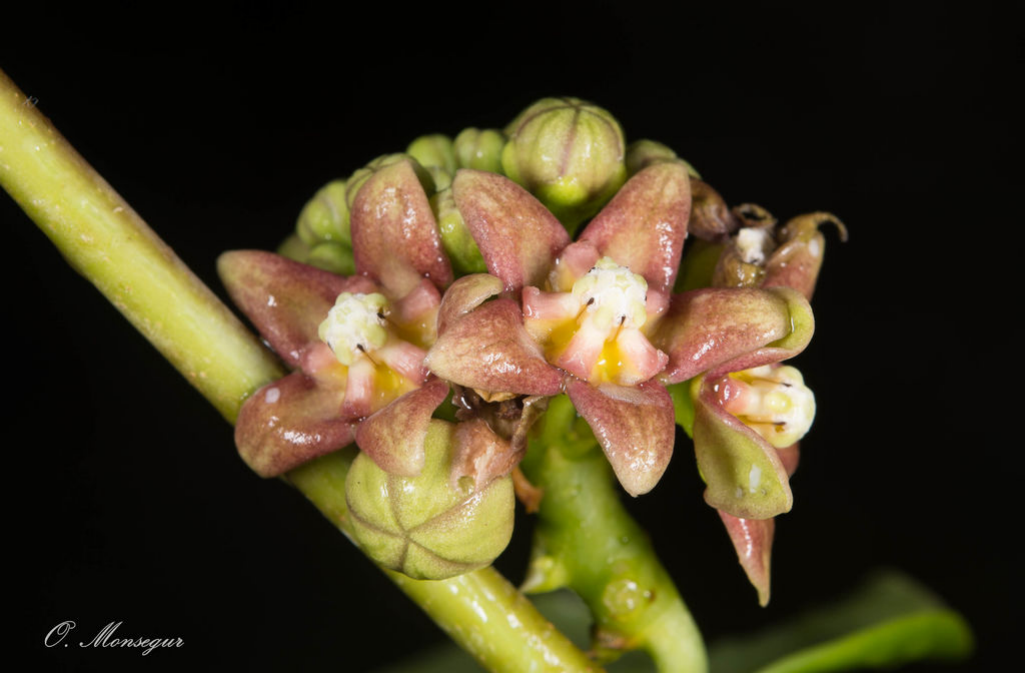
Flowers of specimen of *Ruehssia woodburyana* observed and collected on Norman Island, British Virgin Islands.

**Figure 2.**
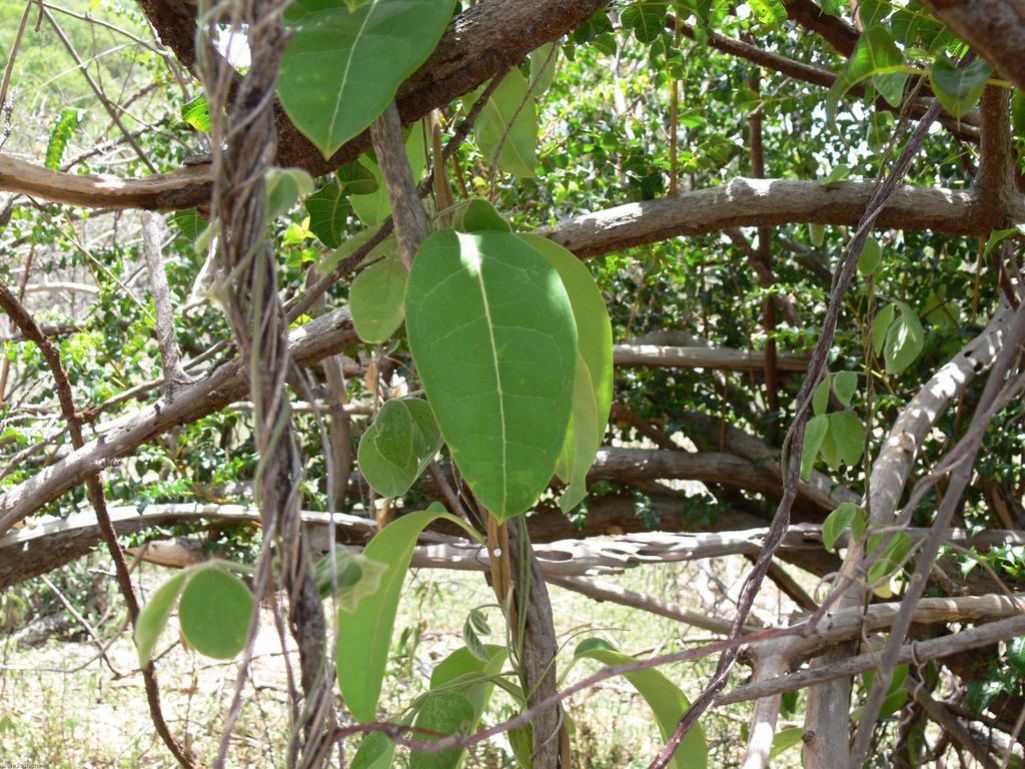
Habit (vine) of *Ruehssia woodburyana* observed in Guánica State Forest, Puerto Rico.

**Figure 3.**
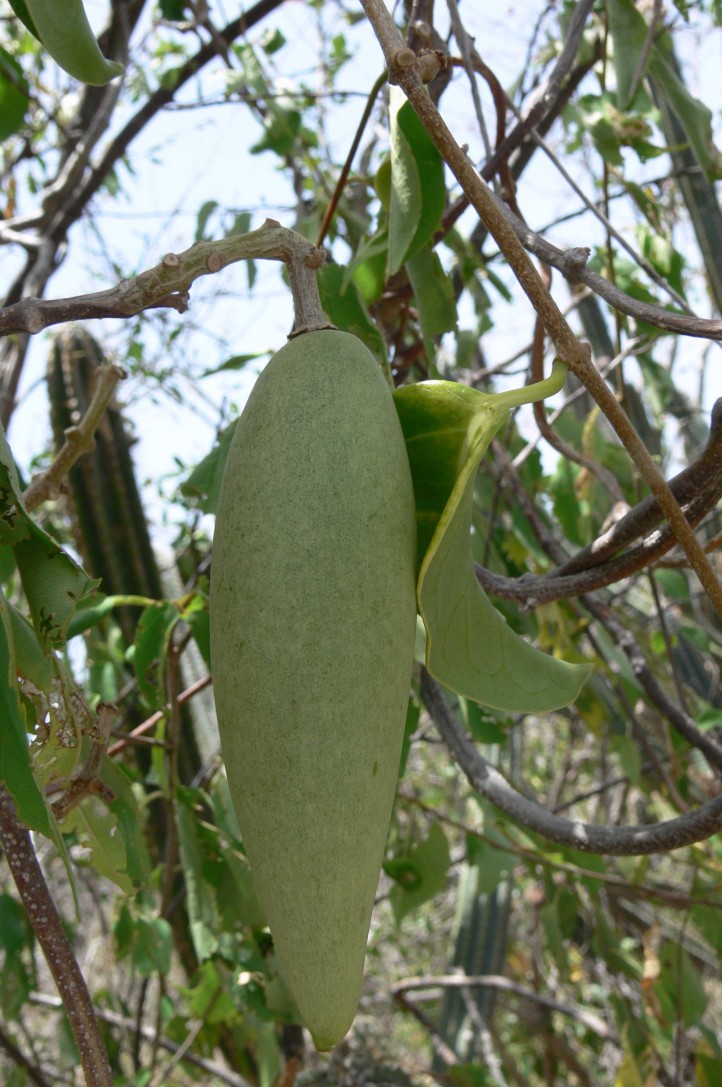
Fruit of *Ruehssia woodburyana* observed in Guánica State Forest, Puerto Rico.

Habitat (narrative): A woody vine which can grow to eight metres long in tropical dry forest (Figs 1, 2, 3, Acevedo-Rodríguez 2005). This species is known to flower only once per year for a short period (Segarra et al. 2014).

Trend in extent, area or quality?: Decline (observed)

##### Habitat

Habitat importance: Major Importance

Habitats: 1.5. Forest - Subtropical/Tropical Dry

#### Habitat

Habitat importance: Major Importance

Habitats: 1.5. Forest - Subtropical/Tropical Dry

#### Ecology

Generation length (yr): 0

Dependency of single sp?: No

Ecology and traits (narrative): The generation length of this vine is unknown. More field observations are required.

#### Threats

Justification for threats: This species is subjected to a variety of threats. Most locations are threatened by human disturbance which is causing habitat degradation and fragmentation, particularly through urban development and fire. In Puerto Rico, human-induced fires are frequent in Guánica State Forest along road PR 333 near Caña Gorda, the type locality. These seriously affect the quality of this species suitable habitat and may preclude the species natural recruitment. The habitat on Norman Island in the BVI was degraded by feral animals in the past, but these have now been removed, promoting the recovery of native vegetation. Despite the presence of feral goats and pigs on Mona Island, these animals are not thought to be impacting the species as feral mammal populations are managed through sports hunting. However, it is noted that no recruitment has been observed at this location in the recent years (J. Sustache pers. comm. 2018). At Laguna Cartagena and Cabo Rojo National Wildlife Refuges, there is no direct evidence of impact due to human-induced fires or feral animals, despite the presence of these threats. Within the municipality of Peñuelas, this species suitable habitat is threatened by the expansion of industrial landfills, service roads and utility lines (O. Monsegur pers. comm. 2018). Climate change might already be impacting this species through more severe droughts and stronger tropical storms.

##### Threats

Threat type: Ongoing

Threats: 1.3. Residential & commercial development - Tourism & recreation areas4.1. Transportation & service corridors - Roads & railroads4.2. Transportation & service corridors - Utility & service lines7.1. Natural system modifications - Fire & fire suppression11.2. Climate change & severe weather - Droughts

#### Threats

Threat type: Ongoing

Threats: 1.3. Residential & commercial development - Tourism & recreation areas4.1. Transportation & service corridors - Roads & railroads4.2. Transportation & service corridors - Utility & service lines7.1. Natural system modifications - Fire & fire suppression11.2. Climate change & severe weather - Droughts

#### Conservation

Justification for conservation actions: This species is found within protected areas across its natural range. In the Commonwealth of Puerto Rico, the species is recorded as occurring within the Guánica State Forest, Laguna Cartagena National Wildlife Refuge, Cabo Rojo National Wildlife Refuge and Mona Island Nature Reserve. It is also thought to occur within the Culebra National Wildlife Reserve, but further surveys are needed. Norman Island in the BVI is not a protected area, as it is privately owned. This species is listed as a Critical Element by the Department of Natural and Environmental Resources (DNER 2007). Monsegur (Monsegur 2009) suggests that this species should also be evaluated to be listed under the U.S. Endangered Species Act of 1973. There are no known *ex situ* collections for this species despite attempts in recent years to collect seed from southern municipalities on the island of Puerto Rico (J. Sustache pers. comm. 2018).

##### Conservation actions

Conservation action type: Needed

Conservation actions: 1.2. Land/water protection - Resource & habitat protection3.4. Species management - Ex-situ conservation4.3. Education & awareness - Awareness & communications

#### Conservation actions

Conservation action type: Needed

Conservation actions: 1.2. Land/water protection - Resource & habitat protection3.4. Species management - Ex-situ conservation4.3. Education & awareness - Awareness & communications

#### Other

Justification for use and trade: There are no known uses for this species.

Justification for ecosystem services : Insufficient Information available

##### Use and trade

Use type: International

##### Ecosystem services

Ecosystem service type: Less important

##### Research needed

Research needed: 1.2. Research - Population size, distribution & trends1.3. Research - Life history & ecology3.4. Monitoring - Habitat trends

Justification for research needed: Conservation action and research should be directed to develop a better understanding of this species' ecology and population trends and develop *ex situ* conservation collections. Further surveys are needed to look for potential undetected individuals and subpopulations within the species range, particularly in the US and British Virgin Islands. The areas and habitats, where this species occurs, should be closely managed and monitored.

#### Use and trade

Use type: International

#### Ecosystem services

Ecosystem service type: Less important

#### Research needed

Research needed: 1.2. Research - Population size, distribution & trends1.3. Research - Life history & ecology3.4. Monitoring - Habitat trends

Justification for research needed: Conservation action and research should be directed to develop a better understanding of this species' ecology and population trends and develop *ex situ* conservation collections. Further surveys are needed to look for potential undetected individuals and subpopulations within the species range, particularly in the US and British Virgin Islands. The areas and habitats, where this species occurs, should be closely managed and monitored.

#### Viability analysis

## Supplementary Material

6355BA10-D648-52AF-8F97-9E52B988512B 10.3897/BDJ.7.e47110.suppl1 Supplementary material 1 Google Earth map showing *Ruehssia woodburyana* known records Data type: occurences
File: oo_307399.kml https://binary.pensoft.net/file/307399 Barrios, S.; Hamilton, H.; Monsegur, O.; Sustache, J.

## Hosting institution

National Parks Trust of the Virgin Island
